# Epidemiology of *Staphylococcus aureus* in neonates on admission to a Chinese neonatal intensive care unit

**DOI:** 10.1371/journal.pone.0211845

**Published:** 2020-02-13

**Authors:** Wenjing Geng, Yujie Qi, Wenting Li, Thomas H. McConville, Alexandra Hill-Ricciuti, Emily C. Grohs, Lisa Saiman, Anne-Catrin Uhlemann

**Affiliations:** 1 Neonatal Center, Beijing Children's Hospital, Capital Medical University, National Center for Children's Health, Beijing, China; 2 Respiratory Department, Qilu Children’s Hospital of Shandong University, Jinan, China; 3 Division of Infectious Diseases, Department of Medicine, Columbia University Medical Center, New York, NY, United States of America; 4 Department of Pediatrics, Columbia University Irving Medical Center, New York, NY, United States of America; 5 Department of Infection Prevention and Control, NewYork-Presbyterian Hospital, New York, NY, United States of America; Instituto de Technologia Quimica e Biologica, PORTUGAL

## Abstract

**Purpose:**

Little is known about the molecular epidemiology of *Staphylococcus aureus* in Chinese neonatal intensive care units (NICUs). We describe the molecular epidemiology of *S*. *aureus* isolated from neonates on admission to Beijing Children's Hospital.

**Methods:**

From May 2015-March 2016, nasal swabs were obtained on admission from 536 neonates. Cultures were also obtained from body sites with suspected infections. *S*. *aureus* isolates were characterized by staphylococcal chromosomal cassette (SCC*mec*) type, staphylococcal protein A (*spa*) type, multilocus sequence type (MLST), *sasX* gene, antimicrobial susceptibility and cytotoxicity. Logistic regression assessed risk factors for colonization.

**Results:**

Overall, 92 (17%) infants were colonized with *S*. *aureus* and 20 (3.7%) were diagnosed with culture-positive *S*. *aureus* infection. Of the colonized infants, 70% (64/92) harbored methicillin-susceptible *S*. *aureus* (MSSA), 30% (28/92) harbored methicillin-resistant *S*. *aureus* (MRSA) while 70% (14/20) of infected infants were culture-positive for MRSA, 30% (6/20) were culture-positive for MSSA. Risk factors for colonization included female sex, age 7–28 days, higher birthweight (3270 IQR [2020–3655] grams) and vaginal delivery (*p*<0.05). The most common MRSA and MSSA clones were community-associated ST59-SCC*mec*IVa-t437 (60%) and ST188-t189 (15%), respectively. The *sasX* gene was not detected. Some MSSA isolates (16%) were penicillin-susceptible and some MRSA isolates (18%) were oxacillin-susceptible. MRSA and MSSA had similar cytotoxicity, but colonizing strains were less cytotoxic than strains associated with infections.

**Conclusions:**

*S*. *aureus* colonization was common in infants admitted to our NICU and two community-associated clones predominated. Several non-modifiable risk factors for *S*. *aureus* colonization were identified. These results suggest that screening infants for *S*. *aureus* upon admission and targeting decolonization of high-risk infants and/or those colonized with high-risk clones could be useful to prevent transmission.

## Introduction

*Staphylococcus aureus* infections represent a significant clinical burden for infants worldwide and were recently found to be the second most common cause of late-onset sepsis in very-low birth weight (VLBW) infants admitted to neonatal intensive care units (NICUs) in the United States and United Kingdom [1, 2]. Preterm infants are also at high risk for *S*. *aureus* colonization [3], a potential risk factor for subsequent infection. In a recent meta-analysis involving patients admitted to NICUs and intensive care units (ICUs), methicillin-resistant *S*. *aureus* (MRSA) colonization was associated with a 24.2 times increased MRSA infection risk [4]. Endemic transmission and outbreaks due to MRSA and methicillin-susceptible *S*. *aureus* (MSSA) occur frequently in NICUs [5]. Studying the molecular epidemiology and virulence factors of *S*. *aureus* in the NICU population can promote an increased understanding of pathogenesis and ultimately guide preventive strategies.

The molecular characteristics of and risk factors for *S*. *aureus* colonization and infection have been described for NICU populations across the globe and have increased our knowledge of the global burden [3, 6]. However, the molecular characteristics of MSSA and MRSA strains isolated from neonates in NICUs in Mainland China have been incompletely described and important gaps in knowledge remain. Existing studies have focused mainly on MRSA in infants or neonates [7–9], whereas little is known about MSSA colonization and infection risks. Li et al. examined neonates who were MRSA positive from different hospitals. Huang YC et al. reported that more than 40% of hospitalized infants were colonized with MRSA during their stay in NICUs and 93% of the colonized neonates were infected with an indistinguishable MRSA isolate [7–9]. Many tertiary NICUs in China are part of dedicated hospitals for children; neonates (less than 28 days old) served by these units are mostly admitted from home after presenting as outpatients. Moreover, the structural layout, patient population, and visiting policies for parents/guardians may differ substantially from NICUs in other countries. For example, in the NICU of Beijing Children’s Hospital (BCH), parents are not allowed to visit and rooms contain four to eight neonates. Therefore, dedicated studies examining the molecular epidemiology of both MSSA and MRSA in Chinese NICUs are needed to improve infection prevention efforts.

In this study, we aimed to determine the proportion of neonates colonized and/or infected with MSSA and MRSA on admission to the NICU of BCH, as well as assess risk factors for *S*. *aureus* colonization. We further aimed to describe the molecular epidemiology of both MSSA and MRSA, including the most dominant clones, and their *in vitro* cytotoxicity. Ultimately, we will use these data to inform future surveillance and *S*. *aureus* prevention efforts.

## Methods

### Study design, study population, and site

From May 2015 to March 2016, we performed a prospective surveillance study on admission for MSSA and MRSA among neonates ≤28 days of age hospitalized in the level 3, 50-bed NICU of BCH. This hospital does not have an obstetrics unit; therefore, neonates (~700–750 annually) are admitted to the NICU from home or other obstetric units. The most common admitting diagnoses are infectious diseases (~60%), prematurity (~20%), and various congenital comorbidities including cardiac, gastrointestinal, and neurologic disorders (~15%). The Ethics Committee of BCH, affiliated with Capital Medical University, approved this study; parents and/or legal guardians of infants provided written informed consent, and none refused participation.

### Demographic and clinical data collection

Selected demographic (e.g., sex, age, delivery type, birthweight) and clinical characteristics (e.g., congenital disease, respiratory support and previous antibiotic use) were abstracted from the electronic medical records of enrolled neonates. Medical records were transferred from their delivery hospital. Age was trichotomized as <7 days, 7–14, and15-28 days of life for analysis, which was consistent with the way neonates have been previously defined [10, 11]. Antibiotic exposure was defined as use of intravenous or oral antibiotics within the seven days prior to admission. Respiratory support was defined as the use of nasal continuous positive airway pressure (NCPAP) or mechanical ventilation within 24 hours of admission. Additionally, diagnoses of suspected infections as described in the NICU admission notes, were also abstracted.

### Surveillance and clinical specimen collection

To detect *S*. *aureus* colonization in the study cohort, both anterior nares were swabbed within 24 hours of admission, following a standard operational procedure. BBL^™^ Culture Swab^™^ Collection and Transport System (Made by Copan for Becton, Dickinson, and Company, Sparks, USA) was used. Specifically, only one swab was used for both nares. The swab was pre-moistened with sterile saline and was inserted in the nasal vestibule, introducing only the cotton part of the swab. The operator rotated the swab while circulating in the nasal vestibule for approximately 5 seconds. This procedure had to be repeated in both nares.

Infants showing suggestive clinical symptoms were considered infected if *S*. *aureus* was isolated from either a normally sterile site (e.g., blood) or cultures obtained for clinical purposes (e.g., skin or eyes). The Clinical Microbiology Laboratory at BCH processed both surveillance and clinical specimens. *S*. *aureus* was identified based on colony morphology and the coagulase test (Saibaisheng, Beijing, China). PCR was used to detect the *mecA* gene [12]. Isolates with zone sizes less than 21 mm for cefoxitin discs (Sigma, USA), according to the criteria of Clinical and Laboratory Standards Institute (CLSI) [13], and which were also *mecA* gene positive were considered MRSA. All *S*. *aureus* isolates were stored at -20° C.

### Molecular-typing and *sasX* detection

For molecular studies, isolates were cultured on blood agar overnight at 37°C. DNA was extracted and used to perform staphylococcal cassette chromosome *mec* (SCC*mec*) typing [14], multi-locus sequence typing (MLST), and staphylococcal protein A (*spa*) typing [15]. The *spa* types were assigned using the Ridom Staph Database (Ridom, Germany) [15]. Sequence types (STs) were assigned using the MLST database (https://pubmlst.org/saureus/) [16]. Additionally, PCR was used to detect the presence of genes encoding for the surface protein *sasX* [17] and for the Panton Valentine leucocidine (PVL), respectively, in all isolates [7].

### Cytotoxicity assays

We prepared *S*. *aureus* culture filtrate preparations, which we obtained from early logarithmic-phase growth (6 hours of incubation). These were used to assess cytotoxicity, as described previously [18]. In brief, *S*. *aureus* isolates were grown in 96-well, round bottom plates in tryptic soy broth for 16–18 hours with shaking at 37°C. Cultures were then diluted 1:75 with fresh Roswell Park Memorial Institute with casamino acids, and 150 μL of the diluted culture was regrown in 96-well, round-bottomed plates for 6 hours at 37°C. Plates were centrifuged and culture supernatants were collected and stored at −80°C for future use. We assayed cytotoxic activity of the culture supernatants using the human myeloid cell line HL-60, differentiated into neutrophil-like cells (PMN-HL60). These have been shown to mimic the sensitivity of human neutrophils to *S*. *aureus*. Twenty microliters of the *S*. *aureus* supernatant were incubated with approximately 1.0×10^5^ PMN-HL60 cells in a final volume of 100 μL (20% v/v supernatants) for 2 hours at 37°C, followed by 2-hour incubation with the CellTiter reagent, monitoring metabolic activity. This dye stains living cells brown, and the intensity of binding was measured in a spectrophotometer at 480nM. Values were compared to positive and negative controls (positive control for cell death = triton x; and negative control for lysis = phosphate buffer saline (PBS)). We assayed each sample in triplicate and independently repeated the assay for each isolate at least twice. If >10% variation was observed between triplicate samples, we repeated the assay.

### Antimicrobial susceptibility testing

In addition to cefoxitin testing carried out by disc diffusion as outlined above, antimicrobial susceptibility to other antibiotics was determined by the agar dilution method, in accordance with the CLSI [13]. MRSA and MSSA isolates were tested for susceptibility to penicillin, oxacillin, gentamicin, ceftriaxone, rifampin, sulfamethoxazole-trimethoprim (TMP-SMX), erythromycin, mupirocin, and levofloxacin. MRSA was also tested for susceptibility to vancomycin, linezolid, fusidic acid, and tigecycline. *S*. *aureus* ATCC 29213 was used as the quality control.

### Statistical analysis

The characteristics of colonized and non-colonized neonates were compared using Chi-squared and Fisher’s exact tests, as appropriate. Colonized infants with concurrent infections were excluded from this analysis. Continuous variables, such as birthweight, were assessed using Mann Whitney U test.

Categorical risk factors for *S*. *aureus* colonization were assessed by comparing the characteristics of colonized vs. non-colonized neonates. For this analysis, birthweight, in grams, was categorized into quartiles (e.g., ≤2600, 2601–3200, 3201–3500, and >3500 grams). Factors with *p*<0.10 in bivariate analysis were then assessed in a multivariate logistic regression model to determine risk factors for overall *S*. *aureus* colonization. To determine independent risk factors for MRSA or MSSA colonization, a multivariate multinomial logistic regression model was used. All statistical tests were two-sided and performed in SAS 9.4 (Cary, NC); a *p*-value<0.05 was considered significant.

Cytotoxicity was expressed as the percentage of cells killed, and the median was compared among MRSA versus MSSA isolates, isolates associated with infections versus colonization, and among the most common STs. Cytotoxicity analyses were performed in GraphPad Prism 7.04 (GraphPad Software, La Jolla, CA) using the Mann Whitney U test.

## Results

### Demographic and clinical characteristics of study population

From May 2015 to March 2016, 536 hospitalized neonates were admitted to the BCH NICU, most of whom (520/536, 97%) were admitted from home after presenting as outpatients, another 16 neonates were transferred from other obstetric units within 24 hours of birth. All were swabbed for *S*. *aureus* nasal colonization on admission. Overall, 17% (n = 92) of the 536 neonates had nasal colonization with *S*. *aureus*, 12% (n = 64) were colonized with MSSA and 5.2% (n = 28) were colonized with MRSA respectively. Four neonates were both colonized and infected. As previous work has shown differences in risk factors between *S*. *aureus* colonization and infection [19] and to avoid misclassification we only considered these four infants in the infection but not the colonization risk factor analyses (n = 88). Colonized infants had a median chronological age at admission of 13 (IQR [8–21.5]) days. When breaking the age into <7,8–14, and 15–28 day categories an association was detected between age and colonization. Male and female infants had similar ages at admission (mean 15 vs. 14 days, respectively, p = 0.40). Colonized infants had significantly higher median birthweights (3285 IQR [3030–3660] grams) than non-colonized infants (3100 IQR [2500–3500] grams, *p* = 0.001).

In this cohort, 255 infants were admitted with suspected infections; of these 20 (7.8%) were culture-positive for *S*. *aureus*. Of the 20 *S*. *aureus* infections, 70% (n = 14) were infected with MRSA and 30% (n = 6) were infected with MSSA. Of the 20 infants who were culture-positive for *S*. *aureus*, one infant had two isolates from the eye and conjunctival secretions, respectively, and another infant had three isolates from the eye, conjunctival secretions, and sputum. Conjunctivitis (n = 8, 35%) and omphalitis (n = 8, 35%) were the most commonly diagnosed *S*. *aureus* infections, followed by pneumonia (n = 3, 13%), impetigo (n = 2, 8.7%), cellulitis (n = 1, 4.3%), and septicemia (n = 1, 4.3%), with 3 infants having more than one site of infection. Four infected neonates were also colonized (2 with MSSA and 2 with MRSA).

### Risk factors for MSSA and MRSA colonization

Risk factors for *S*. *aureus* colonization are shown in Table 1. In the multivariable adjusted logistic regression model, female sex, age, birthweight, and vaginal delivery were associated with *S*. *aureus* colonization, while antibiotic use in the week prior to admission was protective. Newborn provenance (e.g., home vs. hospital transfer) was not associated with colonization. An additional adjusted logistic regression model assessing interaction between birthweight quartiles and neonate age was assessed, but the interaction term was not significant (*p*>0.05).

**Table 1:** Risk factors for *S*. *aureus* colonization in neonates admitted to the NICU of Beijing Children’s Hospital.

Characteristics			Total^1^ (N = 516)	Colonized^1^ (N = 88)	Non-colonized (N = 428)	Crude p-value	OR_ADJ_ (CI_95_)^2^	Adj. p-value
			N (% of population or subpopulation)				
**Demographic**								
	**Sex**					**0.007**	2.00 (1.19, 3.39)	**0.0090**
		Female	200 (39%)	44 (50%)	156 (36%)			
		Male	316 (61%)	44 (50%)	272 (64%)			
		**Birthweight (g)**				**0.0003**		**0.0363**
		<2600	128 (25%)	8 (29%)	120 (28%)		1.00 (Referent)	
		2600–3199	127 (25%)	20 (24%)	107 (25%)		1.70 (0.67–4.28)	
		3200–3500	133 (26%)	31 (25%)	102 (24%)		3.11 (1.29, 7.48)	
		≥3501	128 (25%)	29 (33%)	99 (23%)		2.12 (1.16, 6.98)	
	**Age (days)**					**<0.0001**		**<0.0001**
		<7	241 (47%)	19 (22%)	222 (52%)		1.00 (Referent)	
		7–14	170 (33%)	29 (33%)	141 (33%)		3.31 (1.69–6.49)	
		15–28	105 (20%)	40 (45%)	65 (15%)		10.04 (5.03–20.01)	
	**Newborn Provenance**					0.14	--	--
		Home	502 (97%)	88 (100%)	414 (97%)			
		Transfer	14 (2.7%)	0 (0%)	14 (3.2%)			
	**Delivery type**					**0.0023**	2.12 (1.24, 3.62)	**0.0058**
		Vaginal	251 (49%)	56 (64%)	195 (46%)			
		Cesarean	265 (51%)	32 (36%)	233 (54%)			
**Clinical**								
	**Congenital Disease**					0.53	**--**	**--**
		Yes	86 (17%)	12 (14%)	74 (17%)			
		No	430 (83%)	76 (86%)	354 (83%)			
	**Antibiotic Exposure, prior week**					**0.0024**	0.23 (0.13, 0.43)	**<0.0001**
		Yes	190 (37%)	20 (23%)	170 (40%)			
		No	326 (63%)	68 (77%)	258 (60%)			
	**Respiratory Support**					0.91	--	--
		Yes	96 (19%)	16 (18%)	80 (19%)			
		No	420 (81%)	72 (82%)	348 (81%)			

^1^Excludes any neonate culture positive for infection upon admission (n = 20), including 4 neonates who were both colonized and infected

^2^ Multivariable logistic regression model adjusted for sex, age, delivery, birthweight (quartiles), and antibiotic use

Abbreviations used in table: OR_ADJ_ = Adjusted Odds Ratio, CI_95_ = 95% Confidence Interval

In multinomial logistic regression, female sex (*p* = 0.02), vaginal delivery (*p*<0.0001), and age days (*p*<0.0001) remained significant risk factors for MSSA colonization, while female sex (*p* = 0.02) and age (*p* = 0.001) remained significant risk factors for MRSA colonization. Antibiotic use remained protective for both MSSA and MRSA (*p*<0.0001 and *p* = 0.003, respectively), while birthweight was not associated with either MSSA (*p* = 0.18) or MRSA (*p* = 0.1) colonization.

### Molecular characteristics of *S*. *aureus* isolates

**Fig 1:**
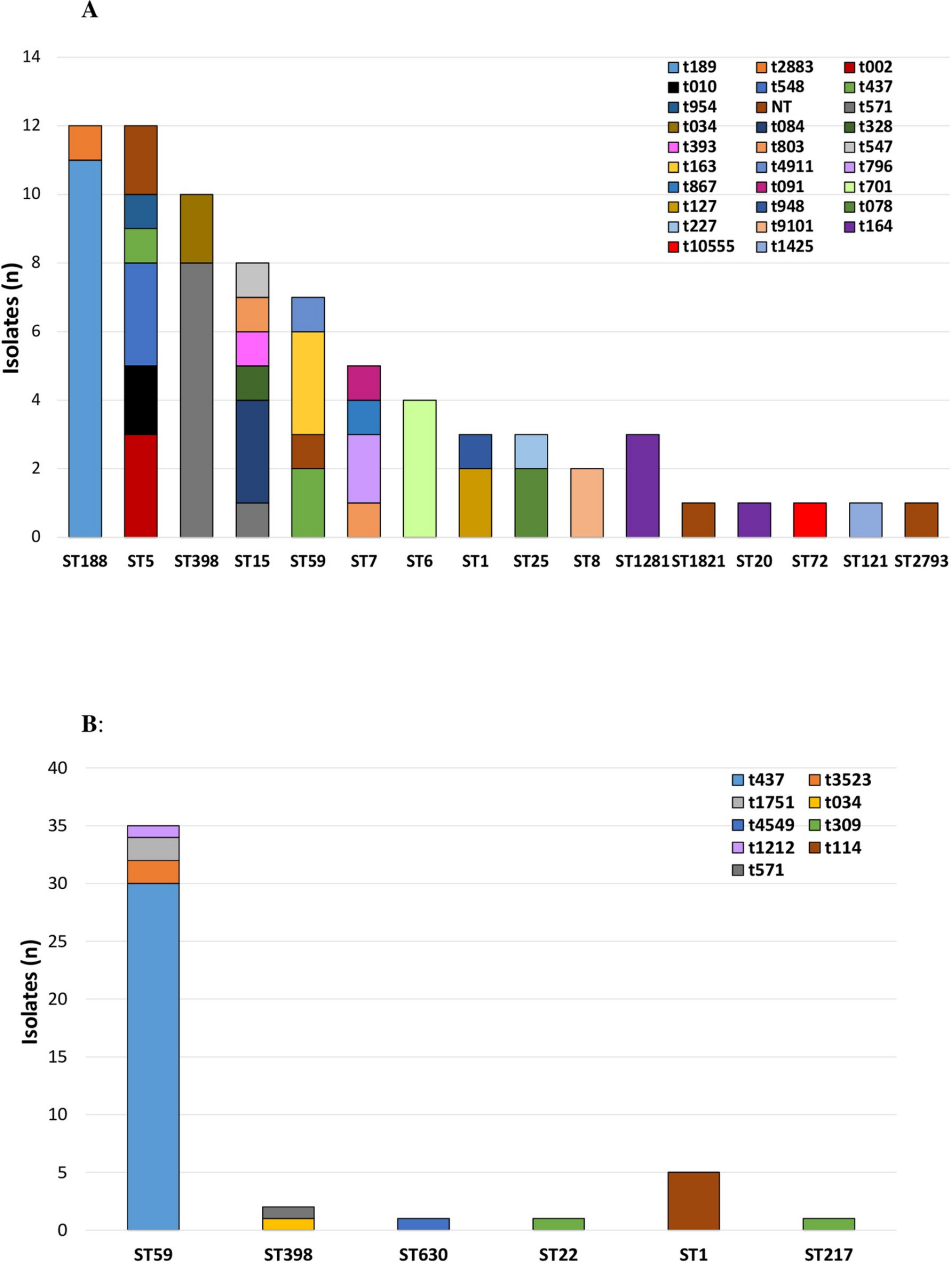
**A.** Distribution of MSSA s*pa* types among sequence types (ST) in neonates admitted to the NICU of Beijing Children’s Hospital, May 2015-March 2016. Overall, 16 STs and 29 spa types were identified in 74 MSSA isolates. The most common STs were ST188 (n = 12, 16%) and ST398 (n = 10, 14%). Abbreviations used in figure: (MSSA, methicillin-susceptible *Staphylococcus aureus*; MLST, multi-locus sequence typing; ST, sequence type). **B.** Distribution of MRSA s*pa* types among sequence types (ST) in neonates admitted to the NICU of Beijing Children’s Hospital, May 2015-March 2016. Overall, 6 STs and 9 *spa-*types were identified in 45 MRSA isolates. The most common ST was ST59 (n = 35, 78%). Abbreviations used in figure: (MSSA, methicillin-susceptible *Staphylococcus aureus*; MLST, multi-locus sequence typing; ST, sequence type).

MLST revealed 16 different sequence types (STs) among the 74 MSSA isolates (Fig 1A), the most common of which were ST188 (n = 12, 16%) and ST5 (n = 12, 16%). Twenty-eight MSSA *spa* types were identified, ST188-t189 (n = 11, 15%) was the most common MSSA clone.

**Fig 2:**
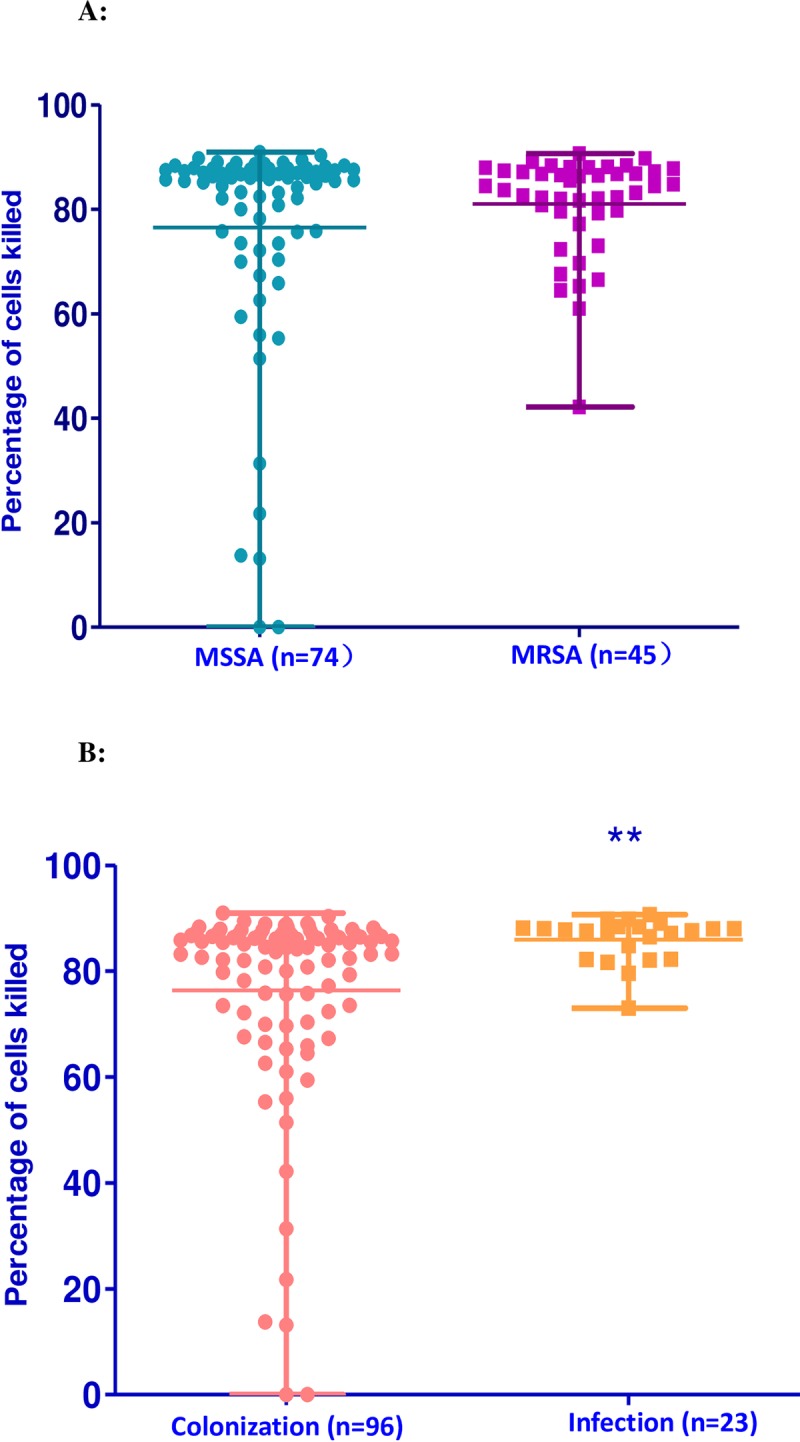
**A.** Cytotoxicity of MSSA versus MRSA isolates obtained from neonates at Beijing Children’s Hospital. The cytotoxicity of MSSA isolates associated with colonization or infection (n = 74) versus MRSA isolates associated with colonization or infection (n = 45) was similar (84 vs 86%, p = 0.85). **B.** Cytotoxicity of MSSA and MRSA isolates associated with colonization versus infection obtained from neonates at Beijing Children’s Hospital. The cytotoxicity of MRSA and MSSA isolates associated with colonization (n = 96) was less than the cytotoxicity of MSSA and MRSA isolates associated with infection (n = 23) (p = 0.008).

MLST revealed six different STs among the MRSA isolates (Fig 1B); ST59 was the most common (n = 35, 78%). Nine MRSA *spa* types were identified, SCC*mec*IVa was the most common SCC*mec* type detected (n = 32, 71%), followed by SCC*mec*V (n = 6, 13%), SCC*mec*IVg (n = 5, 11%), and SCC*mec*III (n = 1, 2.2%); one isolate could not be typed. ST59-SCC*mec*IVa-t437 (n = 27, 60%) was the most common MRSA clone (Fig 2B).

Of 19 *S*. *aureus* isolates associated with skin and soft tissue infections (SSTIs), 3 (17%) belonged to ST398. No MSSA or MRSA isolate harbored *sasX*. The molecular characteristics of the MRSA and MSSA colonizing and infectious isolates are summarized in Table 2 and Table 3.

**Table 2:** Molecular characteristics of MSSA colonizing and infectious isolates in a Chinese NICU.

Colonizing or infectious (NO.)	MLST (NO.)	*spa* (NO.)	PVL positive(NO.)
	ST188(12)	t189 (11)	
		t2883 (1)	
	ST5(11)	t002 (3)	
		t010 (2)	
		t548 (2)	
		t437 (1)	
		t954 (1)	
		NT (2)	
	ST398(8)	t571 (7)	
		t034 (1)	
Colonizing (N = 68)*	ST15 (7)	t084 (1)	1
		t328 (1)	
		t393 (1)	
		t571 (1)	
		t803 (1)	
		t084 (1)	
		t547 (1)	
	ST59 (7)	t163 (3)	
		t437 (2)	
		t4911 (1)	
		NT (1)	
	ST7 (5)	t796 (2)	
		t803(1)	
		t867 (1)	
		t091 (1)	
	ST6 (3)	t701 (3)	
	ST1 (3)	t127 (2)	
		t948 (1)	
	ST25 (3)	t078 (2)	
		t227 (1)	
	ST8 (2)	t9101 (2)	
	ST1281 (2)	t164 (2)	
	ST1821 (1)	NT (1)	
	ST20 (1)	t164 (1)	
	ST72 (1)	t10555 (1)	
	ST121 (1)	t1425 (1)	
	ST2793 (1)	NT (1)	
	ST398 (2)	t571 (1)	2
		t034 (1)	
**Infectious (N = 6)**	ST1281 (1)	t164 (1)	
	ST5 (1)	t548 (1)	
	ST6 (1)	t701 (1)	
	ST15 (1)	t084 (1)	

*From 64 patients

**Table 3:** Molecular characteristics of MRSA colonizing and infectious isolates in a Chinese NICU.

Colonizing or infectious (NO.)	MLST	SCC*mec*(NO.)	*Spa*(NO.)	PVL positive(NO.)
	ST59 (25)	IVa (24)	t437 (20)	6
			t3523 (2)	1
**Colonizing (N = 28)**			t1751 (2)	
		V (1)	t437 (1)	
	ST398 (1)	V (1)	t034 (1)	
	ST630 (1)	III (1)	t4549 (1)	
	ST22 (1)	V (1)	t309 (1)	1
	ST59 (10)	IVa (8)	t437 (7)	7
			t1212 (1)	
		V (1)	t437 (1)	1
Infectious (N = 17)*		NT (1)	t437 (1)	
	ST1 (5)	IVg (5)	t114 (5)	1
	ST398 (1)	V (1)	t571 (1)	
	ST217 (1)	V (1)	t309 (1)	1

*From 14 patients

In this study, there were 4 neonates with both colonization and infection isolates. Genotyping revealed that for 3 pairs, both isolates were identical (same MLST, SCC*mec*, *spa* types). For the 4th pair, we noted a single nucleotide point mutation in the *tpi* gene of MLST (ST217: 7-6-1-5-8-8-6 and ST22: 7-6-1-5-8-5-6).

### Antimicrobial susceptibilities

All 74 MSSA isolates were susceptible to oxacillin, rifampin, TMP-SMX, and mupirocin; 99% (73/74) were also susceptible to levofloxacin, 26% (19/74) to erythromycin and 16% (12/74) to penicillin. All 45 MRSA isolates were susceptible to rifampin, TMP-SMX, mupirocin, levofloxacin, vancomycin, linezolid and tigecycline; 98% (44/45) were also susceptible to gentamicin and fusidic acid (44/45).

Eight MRSA isolates (18%) were oxacillin-susceptible (OS-MRSA), five (63%) of these belonged to ST59-SCC*mec*IVa-t437. Of the 3 other OS-MRSA, one was ST22 and two were ST1. One of the OS-MRSA isolates was recovered from a neonate with infection; the others were from colonization.

### Cytotoxicity of MSSA and MRSA and of colonizing and infectious isolates

**Fig 3:**
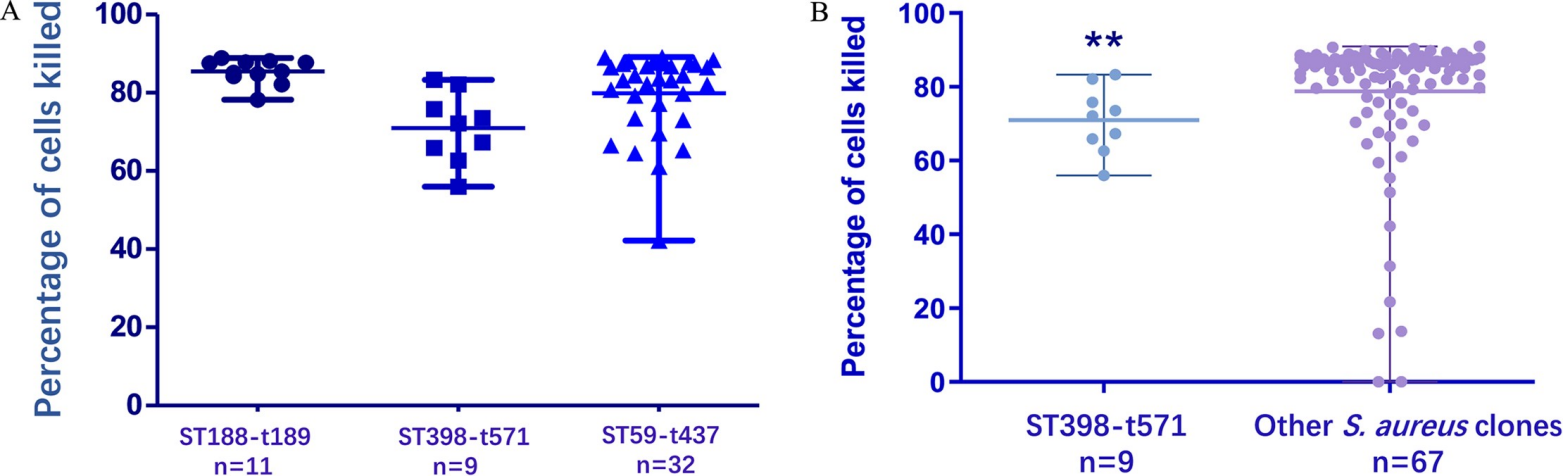
**A.** Cytotoxicity of 3 main *S*. *aureus* clones obtained from neonates at Beijing Children’s Hospital. ST398-t571 isolates (n = 9) had significantly lower cytotoxicity than ST188-t159 (n = 11) and ST59-t437 isolates (n = 32) (*p* = 0.002). **B.** Cytotoxicity of ST398-t571 clones versus all other clones obtained from neonates at Beijing Children’s Hospital.ST398-t571 (n = 9) had significantly lower cytotoxicity when compared to the cytotoxicity of all other clones (n = 67, excluding ST188-t159 and ST59-t437) (*p* = 0.003).

The median cytotoxicity of the 119 isolates was 85% (IQR [76–88%]). We then compared the distribution of data using the Mann Whitney U test between groups. The cytotoxicity of MRSA (median: 84%,) and of MSSA (median: 86%,) were similar (*p* = 0.85) as shown in Fig 2A. The cytotoxicity of the 96 colonizing *S*. *aureus* isolates (median: 85%) was less than that of the 23 infectious isolates (median: 88%), *p* = 0.0008) (Fig 2B). Of the 3 main *S*. *aureus* clones, ST398-t571 had significantly lower cytotoxicity (median: 72%, *p* = 0.002) compared to ST188-t159 (median: 85%) and ST59-t437 (median: 83%; Fig 3A). Additionally, ST398-t571 had significantly lower cytotoxicity when compared to the cytotoxicity of all other clones (median all other clones: 86%, *p* = 0.003, Fig 3B).

The overall prevalence of PVL was 18/45 (40%) of MRSA and 3/74 (4.1%) of MSSA isolates, Most of PVL positive MRSA isolates belonged to ST59-SCC*mec*IVa-t437 (13/18, 72%). The cytotoxicity of the 21 PVL positive isolates (median: 84%) and 98 PVL negative isolates (median: 86%) were similar (p = 0.98).

## Discussion

To our knowledge, this is the first study to assess the burden and molecular epidemiology of *S*. *aureus* at the time of admission to a tertiary care Chinese NICU in Beijing. On admission, 17% of neonates ≤28 days of age had nasal colonization with MSSA or MRSA. This rate was higher than previously reported for neonates within 6 days of birth in Japan (10%) [20], neonates within 1 month of birth in the United States (3.8%) [21], and neonates in a Taiwanese NICU (13%) [22]. In the current study, 5.2% of neonates were colonized with MRSA, which was also a higher rate than previously reported in other NICUs in which rates ranged from 0.3–4.4% [20–22]. Our higher colonization rate may reflect the admission patterns into the NICU of BCH, as 97% of neonates were admitted from home and others were transferred from other obstetric units within 24 hours of birth. An *S*. *aureus* case was considered community acquired if it was isolated from an outpatient or an inpatient within 48 h of hospitalization^14^, so all the *S*. *aureus* were more likely to have been transferred from the community. Furthermore, 7.8% of neonates admitted with suspected infections were culture-positive for *S*. *aureus*, 70% of which were due to MRSA. This contrasts with previous reports in the United States which MSSA represented a greater proportion of *S*. *aureus* infections than MRSA [23]. Our findings demonstrated that admitted neonates continually imported *S*. *aureus* into the NICU, and thus served as potential reservoirs of pathogens for other infants. This suggests that routine surveillance followed by targeted intervention strategies could be useful in reducing *S*. *aureus* infections. However, future studies should assess the effectiveness of surveillance and decolonization in identifying high-risk infants and/ or targeting highly cytotoxic *S*. *aureus* clones in our NICU.

While others have assessed factors associated with *S*. *aureus* colonization in the NICU population, including prematurity and intubation, few have examined the effect of age on colonization risk [4, 6]. We found that neonates aged 7–14 and 15–28 days were at significantly increased risk for both MRSA and MSSA colonization compared with younger neonates. Similarly, Macnow et al found that infants transferred to the NICU at 7 days of age or older had significantly increased odds of colonization with MRSA compared to younger infants, presumably due to more MRSA exposure through interactions with staff, family members, and the healthcare environment [24]. As the age of the neonates and days of initial hospitalization or days at home prior to readmission from initial hospital discharge are not independent we were unable to assess their role in *S*. *aureus* colonization with the current study design. We also found that higher birthweight quartiles were associated with an increased risk of *S*. *aureus* colonization, which contrasts with previous literature [3]; we believe this finding may also be related to the admission patterns of our NICU. In addition, we found that female sex was a risk factor for both MSSA and MRSA colonization, which differed from previous reports. A meta-analysis of risk factors for MRSA colonization in the NICU showed no relationship between colonization and sex [3], while another study from the United States reported male neonates were at increased risk of both MRSA and MSSA colonization [25]. However, our admission patterns did not elucidate an explanation for this finding nor were female neonates older than male neonates on admission. We found vaginal delivery to be a risk factor for MSSA, but not for MRSA colonization. In Shenzhen China, 7.3% vs. 1.7% of pre-partum women were colonized with MSSA and MRSA, respectively, and vaginal delivery was associated with neonatal MSSA, but not with MRSA colonization [25]. Similarly, in a study in New York, Top et al reported MSSA and MRSA anovaginal colonization rates of 11.8% and 0.6%, respectively, in pre-partum women, although neonatal colonization was not assessed [26]. These findings suggest that vertical transmission of *S*. *aureus* occurs, but is more relevant for MSSA than MRSA, presumably because fewer women are colonized with MRSA.

In contrast, neonates who had received antibiotics within seven days of admission were at decreased risk for both MSSA and MRSA colonization. Notably, 188 (37%) neonates had received antibiotics within seven days of admission, as many infants had suspected infections managed as outpatients. Oral antibiotics have not been shown to eradicate MRSA colonization in hospitalized adults [27]. However, the relevance of these studies for neonates in whom the organism burden may be lower or the duration of colonization is likely shorter is uncertain.

MSSA *spa* and STs were diverse, as noted in previous studies [28]. However, ST188 and ST5 were the most common MSSA clones, consistent with previous reports of community-associated MSSA among adults and children in China [29]. ST188 virulence has been in part attributed to epithelial cell adhesion and biofilm formation, properties which could facilitate nasal colonization [29]. Additionally, an American study indicated that livestock-associated MRSA ST5 isolates can adhere to human keratinocytes, which may facilitate colonization with this strain [30]. Importation of common community-associated clones with enhanced adherence properties could potentially facilitate MSSA transmission in the NICU.

ST59-SCC*mec*IVa was the most common MRSA clone, consistent with previous studies of epidemic MRSA clones across Asia [31] and of community-associated MRSA identified in Chinese children’s hospitals [32]. In this study, several MSSA (n = 10) and MRSA (n = 2) isolates belonged to ST398, which was originally reported to colonize livestock and their human handlers [33], but has recently been associated with colonization and infection in distinct human populations in Europe, the Caribbean, and the northeastern United States [34–36]. In China, ST398, is thought to account for as many as 20% of SSTIs caused by *S*. *aureus* [37]. Here, we found that 16% (3/19) of SSTIs caused by *S*. *aureus* were ST398. As previous studies have primarily focused on adult patients, the current study may help elucidate the role of ST398 in *S*. *aureus* infections in the neonatal population.

We also explored the presence of the virulence gene *sasX*, which has been implicated in epidemic spread of MRSA across China and has been demonstrated to play a key role in colonization and pathogenesis, including promotion of immune evasion [38]. However, *sasX* was not detected in the current study, potentially because no neonates harbored ST239, the dominant global healthcare-associated MRSA clone which also predominantly carries *sasX* [38].

Overall, 16% of MSSA strains were penicillin-susceptible. A recent study in Massachusetts found a 3-fold (13% to 32%) increase in penicillin susceptibility among MSSA bloodstream isolates over a decade, speculated to be the result of less selective pressure by β-lactam agents [8]. Future studies should address this possibility in China. Additionally, 18% of MRSA strains were oxacillin-susceptible (OS), which is the first time, to our knowledge, such isolates have been reported in the neonatal population. OS-MRSA is increasingly associated with animal and human infections worldwide [39]; 76% of MRSA isolates from bovine mastitis diagnosed in four Chinese provinces were OS [40]. In our study, ST59-SCC*mec*IV-t437 was the most common OS-MRSA clone (63%), which is consistent with a recent study from China which showed that the most frequent OS-MRSA clones were ST338-t437-SCC*mec*V (32%) and ST59-t437-SCC*mec*IV/V (21%) [41]. Likewise, ST59-t437-SCC*mec*V_T_ was the most prevalent OS-MRSA clone in a study from Taiwan [42]. However, this finding differs from other reports; ST88 and ST8 were the most prevalent OS-MRSA clones in Africa [43] and OS-MRSA clones were highly diverse in Brazil [44], potentially due to geographic differences in genetic background. OS-MRSA strains may be misidentified as MSSA by traditional susceptibility testing thereby complicating the diagnosis and appropriate treatment of *S*. *aureus* infections. Surveillance for such emergent strains should thus be a public health priority.

Furthermore, we found that cytotoxicity of *S*. *aureus* was higher in infectious than in colonizing isolates. Universal decolonization targeting MRSA has been attempted in different healthcare settings to reduce the incidence of infections. These studies have had mixed outcomes. Potential downsides of this approach include the emergence of resistance. Moreover, in many settings MSSA account for higher infection rates than MRSA. We therefore need additional tools to better risk stratify *S*. *aureus* clones that might warrant targeted decolonization, using bacterial determinants other than MRSA status. Given recent investigations into the potential role of virulence ascertained using comparable *in vitro* assays [45] we believe that broader studies need to ascertain the cytotoxicity status of *S*. *aureus* isolates to better understand if these are potentially useful markers to consider in future decolonization programs. We recognize that our study is not designed to fully answer this question; however, we believe that these data provide important insights into the distribution of cytotoxicity of *S*. *aureus* isolates in this high-risk population. Our observations differs from a recent report by Maisem et al. [45], who found an unexpected inverse correlation between *S*. *aureus* toxicity and disease severity when comparing colonizing isolates and those isolated from SSTIs or bacteremia in adult patients. They suggested that bacterial fitness in human serum could explain the unexpected association of low-toxicity isolates with severe, invasive disease. Similarly, Rose et al. [18] reported that low cytotoxic activity and the CC8/239 clone, a weakly cytotoxic lineage, were independent predictors of mortality in MRSA healthcare-associated pneumonia, suggesting that isolates with low cytotoxicity may result in a depressed host response and ultimately worse patient outcomes [18]. We also found that cytotoxicity was linked to the genetic background of *S*. *aureus*, as ST398-t571 exhibited significantly lower cytotoxicity than the two most common clones, as well as all other clones combined. The most common ST398 *spa*-type in our study was t571, associated with human colonization and infection in the US and China [34–36, 46–48], making it less likely that the decreased cytotoxicity reflects an animal-associated *S*. *aureus* background. The association between cytotoxicity, clinical presentations, and outcomes should be studied to further elucidate *S*. *aureus* pathogenesis. Such studies could also have important clinical implications and support targeted rather than universal decolonization of neonates colonized with strains with specific molecular and virulence properties.

Several limitations to our study need to be considered. This was a single NICU cohort study with unique admission patterns, which limits the generalizability of our findings. Since all *S*. *aureus* isolates were collected from neonates upon admission, we could not explore hospital transmission of MSSA or MRSA, nor ascertain whether colonized neonates subsequently developed infections. Surveillance swabs were directly plated onto blood agar plates and did not contain an enrichment step, potentially leading to an underestimation of MRSA carriage. Furthermore, given the study design, we could not assess the potential relationship between cytotoxicity and subsequent infections or clinical outcomes. Because surveillance cultures were only obtained from the nares of neonates and not from other body sites, it is possible that we underestimated the true proportion of colonized neonates. We may have also underestimated infections caused by *S*. *aureus*, as pre-admission antibiotics could have resulted in negative clinical cultures.

In conclusion, the nasal colonization rate of *S*. *aureus* in neonates was high in the NICU of BCH. Female sex, age 7–28 days, higher birthweight and vaginal delivery were risk factors for colonization. While MSSA more frequently colonized neonates, MRSA more frequently infected neonates. Future studies are needed to determine potential sources for MRSA acquisition in these neonates. Most *S*. *aureus* strains were community-associated, reflective of NICU admission patterns. Isolates associated with clinical infection exhibited higher cytotoxicity than colonizing isolates. Our findings suggest that active surveillance of neonates for *S*. *aureus* should be considered as part of strategies to detect importation and prevent transmission of both MRSA and MSSA within the NICU.

## Supporting information

S1 Table (XLSX)

S2 Table (XLSX)

## References

[1] Hornik CP, Fort P, Clark RH, Watt K, Benjamin DK Jr., Smith PB, et al Early and late onset sepsis in very-low-birth-weight infants from a large group of neonatal intensive care units. Early Hum Dev. 2012;88 Suppl 2:S69–74. Epub 2012/05/29. 10.1016/s0378-3782(12)70019-1 22633519 PMC3513766

[2] Cailes B, Kortsalioudaki C, Buttery J, Pattnayak S, Greenough A. Epidemiology of UK neonatal infections: the neonIN infection surveillance network. 2017 10.1136/archdischild-2017-313203 . 29208666

[3] Washam M, Woltmann J, Haberman B, Haslam D, Staat MA. Risk factors for methicillin-resistant *Staphylococcus aureus* colonization in the neonatal intensive care unit: A systematic review and meta-analysis. Am J Infect Control. 2017;45(12):1388–93. Epub 2017/12/03. 10.1016/j.ajic.2017.06.021 . 29195583

[4] Zervou FN, Zacharioudakis IM, Ziakas PD, Mylonakis E. MRSA colonization and risk of infection in the neonatal and pediatric ICU: a meta-analysis. Pediatrics. 2014;133(4):e1015–23. Epub 2014/03/13. 10.1542/peds.2013-3413 . 24616358

[5] Harris SR, Cartwright EJ, Torok ME, Holden MT, Brown NM, Ogilvy-Stuart AL, et al Whole-genome sequencing for analysis of an outbreak of meticillin-resistant *Staphylococcus aureus*: a descriptive study. Lancet Infect Dis. 2013;13(2):130–6. Epub 2012/11/20. 10.1016/S1473-3099(12)70268-2 23158674 PMC3556525

[6] Giuffre M, Amodio E, Bonura C, Geraci DM, Saporito L, Ortolano R, et al Methicillin-resistant *Staphylococcus aureus* nasal colonization in a level III neonatal intensive care unit: Incidence and risk factors. Am J Infect Control. 2015;43(5):476–81. Epub 2015/03/03. 10.1016/j.ajic.2014.12.027 . 25726131

[7] Li S, Ning X, Song W, Dong F, Zheng Y, Chen Q, et al Clinical and molecular characteristics of community-acquired methicillin-resistant *Staphylococcus aureus* infections in Chinese neonates. APMIS. 2015;123(1):28–36. Epub 2014/08/19. 10.1111/apm.12304 . 25132016

[8] Chabot MR, Stefan MS, Friderici J, Schimmel J, Larioza J. Reappearance and treatment of penicillin-susceptible *Staphylococcus aureus* in a tertiary medical centre. J Antimicrob Chemother. 2015;70(12):3353–6. Epub 2015/09/06. 10.1093/jac/dkv270 . 26342027

[9] Huang YC, Chou YH, Su LH, Lien RI, Lin TY. Methicillin-resistant *Staphylococcus aureus* colonization and its association with infection among infants hospitalized in neonatal intensive care units. Pediatrics. 2006;118(2):469–74. Epub 2006/08/03. 10.1542/peds.2006-0254 . 16882797

[10] Lehtonen L, Gimeno A, Parra-Llorca A, Vento M. Early neonatal death: A challenge worldwide. Seminars in fetal & neonatal medicine. 2017;22(3):153–60. Epub 2017/02/28. 10.1016/j.siny.2017.02.006 . 28238633

[11] Oza S, Lawn JE, Hogan DR, Mathers C, Cousens SN. Neonatal cause-of-death estimates for the early and late neonatal periods for 194 countries: 2000–2013. Bulletin of the World Health Organization. 2015;93(1):19–28. Epub 2015/01/06. 10.2471/BLT.14.139790 25558104 PMC4271684

[12] Bignardi GE, Woodford N, Chapman A, Johnson AP, Speller DC. Detection of the *mec*-A gene and phenotypic detection of resistance in *Staphylococcus aureus* isolates with borderline or low-level methicillinresistance. J Antimicrob Chemother. 1996;37(1):53–63. Epub 1996/01/01. 10.1093/jac/37.1.53 . 8647774

[13] CLSI. M100-S25 performance standards for antimicrobial susceptibility testing; Twenty-fifth informational supplement. 2016.

[14] Milheirico C, Oliveira DC, de Lencastre H. Update to the multiplex PCR strategy for assignment of mec element types in *Staphylococcus aureus*. Antimicrob Agents Chemother. 2007;51(9):3374–7. 10.1128/AAC.00275-07 . 17576837 PMC2043198

[15] Koreen L, Ramaswamy SV, Graviss EA, Naidich S, Musser JM, Kreiswirth BN. *spa* typing method for discriminating among *Staphylococcus aureus* isolates: implications for use of a single marker to detect genetic micro- and macrovariation. J Clin Microbiol. 2004;42(2):792–9. Epub 2004/02/10. 10.1128/JCM.42.2.792-799.2004 14766855 PMC344479

[16] Enright MC, Day NP, Davies CE, Peacock SJ, Spratt BG. Multilocus sequence typing for characterization of methicillin- resistant and methicillin-susceptible clones of *Staphylococcus aureus*. J Clin Microbiol. 2000;38(3):1008–15. . 10698988 10.1128/jcm.38.3.1008-1015.2000 PMC86325

[17] Kong H, Fang L, Jiang R, Tong J. Distribution of *sasX*, *pvl*, and *qacA/B* genes in epidemic methicillin-resistant *Staphylococcus aureus* strains isolated from East China. Infect Drug Resist. 2018;11:55–9. Epub 2018/02/02. 10.2147/IDR.S153399 29386909 PMC5765971

[18] Rose HR, Holzman RS, Altman DR, Smyth DS, Wasserman GA, Kafer JM, et al Cytotoxic virulence predicts mortality in nosocomial pneumonia due to methicillin-resistant *Staphylococcus aureus*. J Infect Dis. 2015;211(12):1862–74. Epub 2014/10/10. 10.1093/infdis/jiu554 25298028 PMC4836718

[19] Miller M, Cook HA, Furuya EY, Bhat M, Lee MH, Vavagiakis P, et al Staphylococcus aureus in the community: colonization versus infection. PLoS One. 2009;4(8):e6708 Epub 2009/08/21. 10.1371/journal.pone.0006708 . 19693269 PMC2724739

[20] Mitsuda T, Arai K, Fujita S, Yokota S. Demonstration of mother-to-infant transmission of *Staphylococcus aureus* by pulsed-field gel electrophoresis. Eur J Pediatr 1996;155(3):194–9. Epub 1996/03/01. 10.1007/bf01953937 . 8929727

[21] James L, Gorwitz RJ, Jones RC, Watson JT, Hageman JC, Jernigan DB, et al Methicillin-resistant *Staphylococcus aureus* infections among healthy full-term newborns. Arch Dis Child. 2008;93(1):F40–4. Epub 2007/04/07. 10.1136/adc.2006.104026 . 17412749

[22] Kuo CY, Huang YC, Huang DT, Chi H, Lu CY, Chang LY, et al Prevalence and molecular characterization of *Staphylococcus aureus* colonization among neonatal intensive care units in Taiwan. Neonatology. 2014;105(2):142–8. Epub 2013/12/21. 10.1159/000356733 . 24356303

[23] Carey AJ, Duchon J, Della-Latta P, Saiman L. The epidemiology of methicillin-susceptible and methicillin-resistant *Staphylococcus aureus* in a neonatal intensive care unit, 2000–2007. J Perinatol. 2010;30(2):135–9. Epub 2009/08/28. 10.1038/jp.2009.119 . 19710681

[24] Macnow T, O'Toole D, DeLaMora P, Murray M, Rivera K, Whittier S, et al Utility of surveillance cultures for antimicrobial resistant organisms in infants transferred to the neonatal intensive care unit. Pediatr Infect Dis J. 2013;32(12):e443–50. Epub 2013/07/03. 10.1097/INF.0b013e3182a1d77f . 23811747

[25] Lin J, Wu C, Yan C, Ou Q, Lin D, Zhou J, et al A prospective cohort study of *Staphylococcus aureus* and methicillin-resistant *Staphylococcus aureus* carriage in neonates: the role of maternal carriage and phenotypic and molecular characteristics. Infect Drug Resist. 2018;11:555–65. Epub 2018/05/08. 10.2147/IDR.S157522 29731644 PMC5926071

[26] Top KA, Huard RC, Fox Z, Wu F, Whittier S, Della-Latta P, et al Trends in methicillin-resistant *Staphylococcus aureus* anovaginal colonization in pregnant women in 2005 versus 2009. J Clin Microbiol. 2010;48(10):3675–80. Epub 2010/08/06. 10.1128/JCM.01129-10 20686089 PMC2953117

[27] Loeb MB, Main C, Eady A, Walker-Dilks C. Antimicrobial drugs for treating methicillin-resistant *Staphylococcus aureus* colonization. Cochrane Database Syst Rev. 2003;(4):Cd003340 Epub 2003/10/30. 10.1002/14651858.CD003340 . 14583969 PMC12334153

[28] Asadollahi P, Farahani NN, Mirzaii M, Khoramrooz SS, van Belkum A, Asadollahi K, et al Distribution of the most prevalent *spa* types among clinical isolates of methicillin-resistant and -susceptible *Staphylococcus aureus* around the world: A Review. Front Microbiol. 2018;9:163 Epub 2018/03/01. 10.3389/fmicb.2018.00163 29487578 PMC5816571

[29] Wang Y, Liu Q. Phylogenetic analysis and virulence determinant of the host-adapted *Staphylococcus aureus* lineage ST188 in China. Emerg Microbes Infect. 2018;7(1):45 10.1038/s41426-018-0048-7 . 29593254 PMC5874244

[30] Hau SJ, Kellner S, Eberle KC, Waack U, Brockmeier SL, Haan JS, et al Methicillin-resistant *Staphylococcus aureus* Sequence Type (ST) 5 isolates from health care and agricultural sources adhere equivalently to human keratinocytes. Appl Environ Microbiol. 2018;84(2). Epub 2017/11/05. 10.1128/aem.02073-17 29101193 PMC5752859

[31] Geng W, Yang Y, Wu D, Huang G, Wang C, Deng L, et al Molecular characteristics of community-acquired, methicillin-resistant *Staphylococcus aureus i*solated from Chinese children. FEMS Immunol Med Microbiol. 2010;58(3):356–62. Epub 2010/02/06. 10.1111/j.1574-695X.2010.00648.x . 20132304

[32] Li S, Sun J, Zhang J, Li X, Tao X, Wang L, et al Comparative analysis of the virulence characteristics of epidemic methicillin-resistant *Staphylococcus aureus* (MRSA) strains isolated from Chinese children: ST59 MRSA highly expresses core gene-encoded toxin. APMIS. 2014;122(2):101–14. Epub 2013/05/29. 10.1111/apm.12105 . 23710711

[33] Witte W, Strommenger B, Stanek C, Cuny C. Methicillin-resistant *Staphylococcus aureus* ST398 in humans and animals, Central Europe. Emerg Infect Dis. 2007;13(2):255–8. 10.3201/eid1302.060924 . 17479888 PMC2725865

[34] Bhat M, Dumortier C, Taylor BS, Miller M, Vasquez G, Yunen J, et al *Staphylococcus aureus* ST398, New York City and Dominican Republic. Emerg Infect Dis. 2009;15(2):285–7. Epub 2009/02/06. 10.3201/eid1502.080609 . 19193274 PMC2657615

[35] Uhlemann AC, McAdam PR, Sullivan SB, Knox JR, Khiabanian H, Rabadan R, et al Evolutionary dynamics of pandemic methicillin-sensitive *Staphylococcus aureus* ST398 and its international spread via routes of human migration. MBio. 2017;8(1). 10.1128/mBio.01375-16 28096484 PMC5241395

[36] David MZ, Siegel J, Lowy FD, Zychowski D, Taylor A, Lee CJ, et al Asymptomatic carriage of sequence type 398, *spa* type t571 methicillin-susceptible *Staphylococcus aureus* in an urban jail: a newly emerging, transmissible pathogenic strain. J Clin Microbiol. 2013;51(7):2443–7. 10.1128/JCM.01057-13 23658269 PMC3697676

[37] Zhao C, Liu Y, Zhao M, Liu Y, Yu Y, Chen H, et al Characterization of community acquired *Staphylococcus aureus* associated with skin and soft tissue infection in Beijing: high prevalence of PVL+ ST398. PLoS One. 2012;7(6):e38577 10.1371/journal.pone.0038577 22701673 PMC3368899

[38] Li M, Du X, Villaruz AE, Diep BA, Wang D, Song Y, et al MRSA epidemic linked to a quickly spreading colonization and virulence determinant. Nat Med. 2012;18(5):816–9. 10.1038/nm.2692 22522561 PMC3378817

[39] Pournaras S, Stathopoulos C, Tsakris A. Oxacillin-susceptible MRSA: could it become a successful MRSA type? Future Microbiol. 2013;8(11):1365–7. Epub 2013/11/10. 10.2217/fmb.13.118 . 24199795

[40] Pu W, Su Y, Li J, Li C, Yang Z, Deng H, et al High incidence of oxacillin-susceptible *mec*A-positive *Staphylococcus aureus* (OS-MRSA) associated with bovine mastitis in China. PLoS One. 2014;9(2):e88134 10.1371/journal.pone.0088134 24523877 PMC3921137

[41] Song Y, Cui L, Lv Y, Li Y, Xue F. Characterisation of clinical isolates of oxacillin-susceptible *mec*A-positive *Staphylococcus aureus* in China from 2009 to 2014. J Glob Antimicrob Resist. 2017;11:1–3. Epub 2017/07/22. 10.1016/j.jgar.2017.05.009 . 28729204

[42] Ho CM, Lin CY, Ho MW, Lin HC, Chen CJ, Lin LC, et al Methicillin-resistant *Staphylococcus aureus* isolates with SCC*mec* type V and *spa* types t437 or t1081 associated to discordant susceptibility results between oxacillin and cefoxitin, Central Taiwan. Diagn Microbiol Infect Dis. 2016;86(4):405–11. 10.1016/j.diagmicrobio.2016.08.025 . 27650515

[43] Conceicao T, Coelho C, de Lencastre H, Aires-de-Sousa M. Frequent occurrence of oxacillin-susceptible *mec*A-positive *Staphylococcus aureus* (OS-MRSA) strains in two African countries. J Antimicrob Chemother. 2015;70(12):3200–4. 10.1093/jac/dkv261 . 26318189

[44] Andrade-Figueiredo M, Leal-Balbino TC. Clonal diversity and epidemiological characteristics of *Staphylococcus aureus*: high prevalence of oxacillin-susceptible *mec*A-positive *Staphylococcus aureus* (OS-MRSA) associated with clinical isolates in Brazil. BMC Microbiol. 2016;16(1):115 Epub 2016/06/22. 10.1186/s12866-016-0733-4 27325108 PMC4915036

[45] Laabei M, Uhlemann AC, Lowy FD, Austin ED, Yokoyama M, Ouadi K, et al Evolutionary trade-offs underlie the multi-faceted virulence of *Staphylococcus aureus*. PLoS Biol. 2015;13(9):e1002229 10.1371/journal.pbio.1002229 26331877 PMC4558032

[46] Uhlemann AC, Porcella SF, Trivedi S, Sullivan SB, Hafer C, Kennedy AD, et al Identification of a Highly transmissible animal-independent *Staphylococcus aureus* ST398 clone with distinct genomic and cell adhesion properties. MBio. 2012;3(2). 10.1128/mBio.00027-12 22375071 PMC3302565

[47] Uhlemann AC, Hafer C, Miko BA, Sowash MG, Sullivan SB, Shu Q, et al Emergence of sequence type 398 as a community- and healthcare-associated methicillin-susceptible *Staphylococcus aureus* in northern Manhattan. Clin Infect Dis. 2013;57(5):700–3. cit375 [pii] 10.1093/cid/cit375 23728142 PMC3739468

[48] Yan X, Schouls LM, Pluister GN, Tao X, Yu X, Yin J, et al The population structure of *Staphylococcus aureus* in China and Europe assessed by multiple-locus variable number tandem repeat analysis; clues to geographical origins of emergence and dissemination. Clin Microbiol Infect. 2016;22(1):60 e1–e8. 10.1016/j.cmi.2015.08.022 . 26344334

